# Sero‐Epidemiology and Molecular Detection of Bluetongue Virus in Goats in Bangladesh

**DOI:** 10.1002/vms3.70909

**Published:** 2026-03-31

**Authors:** Md Habibur Rahman, Md Nurul Haque, Md Zulfekar Ali, Lutfun Naher, Md Zakir Hassan, Sadek Ahmed

**Affiliations:** ^1^ Animal Health Research Division Bangladesh Livestock Research Institute Savar, Dhaka Bangladesh; ^2^ Goat Production Research Division Bangladesh Livestock Research Institute Savar, Dhaka Bangladesh

**Keywords:** Bangladesh, bluetongue, goat, nested PCR, risk factors, seroprevalence

## Abstract

**Background:**

Bluetongue (BT) is an arthropod‐transmitted, non‐contagious viral disease of domestic ruminants caused by bluetongue virus (BTV). The incidence of BT in Bangladesh is poorly understood, and there is no molecular evidence of BTV in the country.

**Objectives:**

This study aimed to determine the seroprevalence of BTV, identify associated risk factors and confirm BTV infection in goats in Bangladesh using reverse transcription polymerase chain reaction (RT‐PCR).

**Materials and Methods:**

A total of 460 goat serum samples were randomly collected from ten goat‐rich districts of Bangladesh from July 2023 to June 2024. Farmers were interviewed using a structured questionnaire to determine potential risk factors. Serum samples were screened for anti‐BTV antibodies using competitive enzyme‐linked immunosorbent assay (c‐ELISA), and logistic regression models were applied to identify potential risk factors. Nested RT‐PCR was performed on ELISA‐positive pooled blood samples for molecular detection of BTV using VP7 and NS1 gene‐specific primers.

**Results:**

The overall seroprevalence was 63.04% (95% confidence interval [CI]: 58.63–67.45). Multivariate logistic regression analysis revealed that breed (crossbred; OR: 3.4, 95% CI: 1.34–8.63), sex (female; OR: 1.97, 95% CI: 1.15–3.40), age (over 2 years; OR: 6.32, 95% CI: 2.42–16.81), biosecurity (poor; OR: 26.48, 95% CI: 1.72–405.6), farm type (household; OR: 33.72, 95% CI: 3.02–375.5), flock size (large; OR: 30.53, 95% CI: 2.91–319.6) and vector control (no; OR: 27.56, 95% CI: 8.60–88.28) were the major risk factors associated with the seroprevalence of anti‐BTV antibodies. Nested RT‐PCR successfully amplified the VP7 (770 bp) and NS1 (101 bp) genes. Among the 58 pooled samples (each pool containing 5 of the 290 ELISA‐positive samples), 13 pools (22.4%) tested positive for BTV RNA, confirming the detection of the virus in goats by PCR.

**Conclusion:**

This study provides the first large‐scale evidence of BTV exposure and PCR‐based detection in goats in Bangladesh and highlights important epidemiological risk factors for BTV transmission.

## Graphical abstract

The first large‐scale study in Bangladesh confirmed bluetongue virus infection in goats through combined c‐ELISA and nested RT‐PCR approaches, revealing high seroprevalence and identifying key management‐related risk factors.

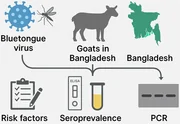

## 1 Introduction

Goats play an important role in global livestock systems, particularly in sustaining the nutritional health of rural populations and landless small‐scale farmers in tropical regions (Nair et al. 2021). They also possess a significant importance in the national economy, serving as an important component for both large‐ and small‐scale farmers in their farming practices. In addition to providing milk and meat as major sources of protein, goats generate income through the sale of live animals, skins and carcasses, thereby contributing significantly to household livelihoods and national trade (Welay et al. 2018). The raising of goats is increasing in Bangladesh (Haque and Rahman 2024) to satisfy the demand for animal protein; however, various diseases and disorders are hindering the growth of this industry (Rahman et al. 2020). The hot and humid climate of Bangladesh fosters diseases that diminish the productivity of animals and increase veterinary costs (Haque and Rahman 2024; Rahman et al. 2024). Goat production is significantly affected by various infectious diseases, with bluetongue (BT) identified as a serious emerging disease in Bangladesh (Rahman et al. 2022). BT is a viral disease affecting goats and sheep, characterized as infectious yet non‐contagious and transmitted by arthropods. Its causal agent is the bluetongue virus (BTV) under *Orbivirus* genus and Sedoreoviridae family (Benelli et al. 2017; Qin et al. 2018). BTV is a virus characterized by the absence of an envelope, featuring ten distinct segments of its double‐stranded RNA (dsRNA) genome, which is encircled with an icosahedral triple stack. The BTV genome encodes five non‐structural proteins (NS1–NS5) and seven structural proteins (VP1–VP7) (Ayanur et al. 2016; White et al. 2019). The VP7 protein of BTV is highly conserved and immunodominant, making it a reliable target for serological assays across diverse BTV serotypes (White et al. 2019; Saminathan et al. 2020). The analysis of the genome segment‐2 (Seg‐2) sequence and its corresponding translated protein VP2 reveals the existence of 28 serotypes of BTV (Saminathan et al. 2020). Its primary mode of transmission involves blood‐feeding insects identified as *Culicoides* spp., which facilitate the spread of the infection to susceptible hosts. This includes both domestic and wild species such as sheep, goats, cattle, buffalo, deer and other members of the Artiodactyla family from infected viremic animals (Belbis et al. 2017; Islam et al. 2023). Cattle and goats frequently serve as asymptomatic carriers of the virus, whereas sheep and deer tend to exhibit clinical signs more readily, with certain instances leading to moderate to high fatality rates (Constable 2017). World Organisation for Animal Health has described BT as a notifiable disease due to its significant morbidity and mortality rates (Saminathan et al. 2020; Hassani and Madadgar 2021).

The primary manifestations of this disease include fever, serous to bloody discharge from the nose, subsequent mucopurulent discharge, hyperaemia and swelling of the lips, face, ears and submaxillary region, resulting in a ‘monkey face’ appearance. Additional symptoms include oral erosions and wounds, coronitis, tongue cyanosis, muscular necrosis and breathing difficulties ultimately resulting in weakness and fatality (Channappanavar et al. 2012; Saminathan et al. 2020). Restrictions on trade that limit access to valuable markets result in passive losses, whereas direct losses arise from production declines, including mortality, abortion and reductions in milk and meat production, as well as costs related to vaccines and control measures (Rushton and Lyons 2015; Gethmann et al. 2020). Worldwide impacts resulting from BT outbreaks were estimated at $3 billion (Ben Salem et al. 2024) and pose a significant risk of economic losses in Bangladesh, because BTV is endemic in India, Myanmar, China and Pakistan (Joardar et al. 2013; Saminathan et al. 2020). Many studies indicate the existence of BT in goats, cattle, camels and buffalo across various states in India adjacent to the Bangladesh border (Joardar et al. 2013; De et al. 2019). BT has also been reported in Afghanistan, Nepal, Pakistan, India and Sri Lanka (Saminathan et al. 2020). Bangladesh has a large bordering area with India to the west, north and east and with Myanmar to the south (Islam et al. 2023). These countries have undertaken many steps to assess the prevalence, identify the causal agent, and implement control and prevention measures for this infection (Reddy et al. 2016; Rao et al. 2017; Joardar 2022).

In Bangladesh, studies on BT are very limited. Serological evidence of BTV was first reported in sheep from the Chattogram district near the Myanmar border, based on a small‐scale survey of 150 samples (Munmun et al. 2022). Another investigation examined both sheep and goats across three districts only; however, the goat sample size was only about 123 (Islam et al. 2023), limiting the representativeness of the findings. Although these studies provided valuable initial insights, they remain fragmentary and insufficient to understand the true prevalence and epidemiological status of BTV in the country. To date, comprehensive epidemiological and molecular data on BT in goats, including associated risk factors, are lacking in Bangladesh. Therefore, a large‐scale serological survey combined with molecular investigations is essential to identify affected regions, improve prediction and strengthen control strategies for this disease. Considering the significant economic impact of BT and the scarcity of reliable scientific data, the present study was undertaken to provide comprehensive epidemiological data, determine risk factors as well as provide molecular confirmation of BTV infection in goats for the first time in Bangladesh. The findings are expected to offer new perspectives on the prevalence and epidemiology of BT, thereby supporting the development of effective control and prevention measures in the country.

## 2 Materials and Methods

### 2.1 Study Population, Areas and Duration

**FIGURE 1:**
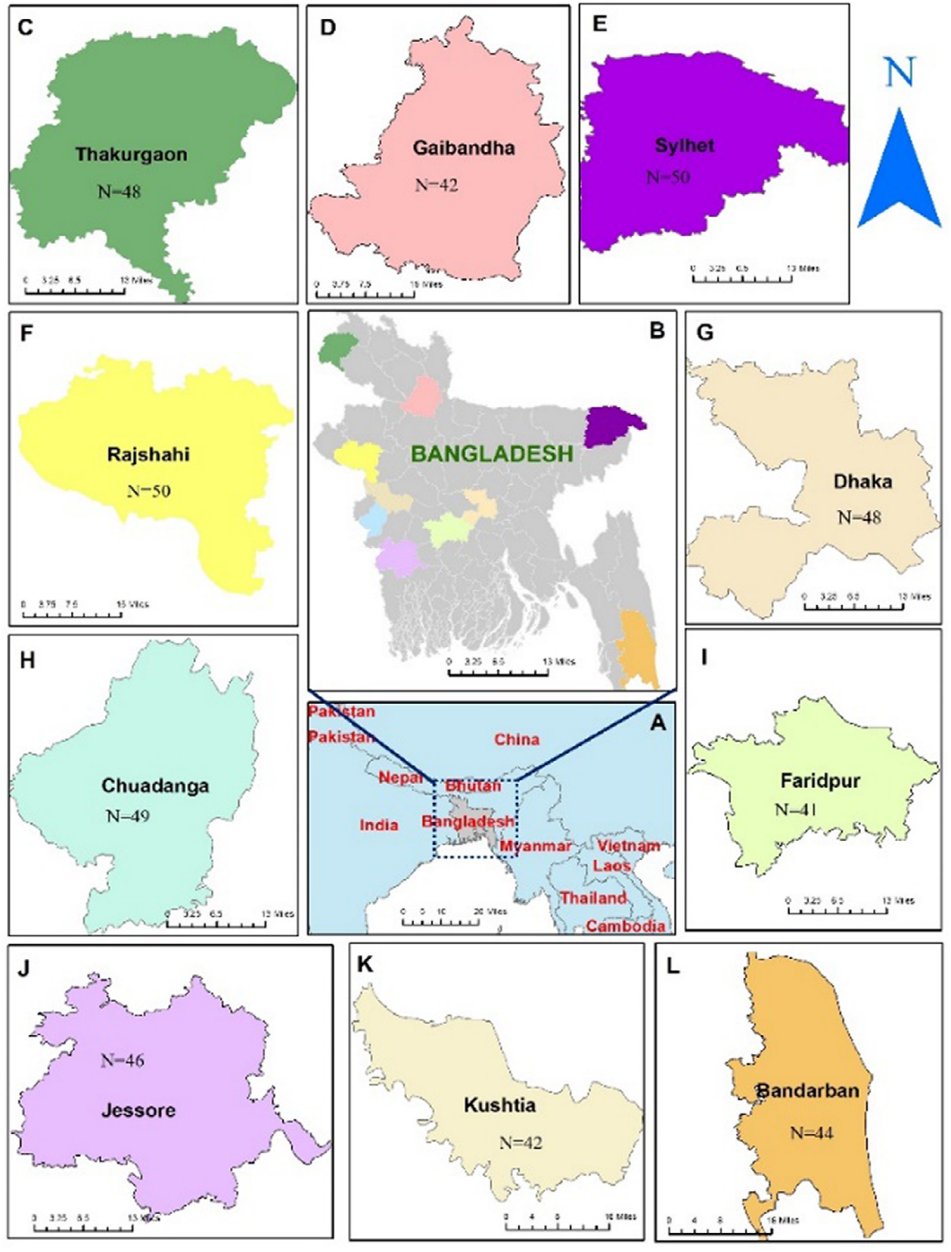
Study areas and collected sample number.

This cross‐sectional study was conducted on goats from 10 goat‐populated districts of Bangladesh between July 2023 and June 2024. A total of 460 blood samples were taken from randomly selected goats living in the study locations (Figure 1). Within each farm, goats were randomly selected on the basis of animal availability, ensuring representation across different age, sex and breed categories, rather than sampling all goats in the herd. Samples comprising Black Bengal (BBG), Jamunapari (JP) and crossbred goats of both sexes were collected from small (1–10 goats), medium (11–50 goats) and large (>50 goats) flocks across three age categories: Under 6 months (kids), 6–24 months (growing) and over 24 months (adults) were taken for the study. Farmers at the research location practiced semi‐intensive and free‐range methods for goat rearing. None of the goats included in this study had been vaccinated against BTV.

### 2.2 Sampling Strategy

As there have been relatively few prior reports on BTV from Bangladesh, we estimated the sample size according to the formula by Thrusfield (2018) using the prevalence data from neighbouring countries and other sources. The outcome factor's percentage frequency in the overall population (p) is 50% in a 95% confidence interval (CI) and 5% desired absolute precision:

$$n\;=\;1.96^{2}P\text{exp}\left(1\;-\;P\text{exp}\right)/d^{2}$$

where *n* is the required sample size, *P*exp is the expected prevalence, and *d* is the desired absolute precision (5%).

To achieve the highest level of precision, 460 goats were sampled out of the 384 needed for this investigation.

### 2.3 Data Collection

Information on goat rearing was obtained using structured and pretested questionnaires (Supporting Information File 1) during direct interviews to the owners or their representatives. When the interview took place, information about the sex, age, breed, housing, raising method, size of the flock, farm type, biosecurity status, knowledge about BTV, vector control, rearing with other species, vaccination against PPR and body condition score (BCS) of goats was obtained (Supporting Information File 2).

### 2.4 Sample Collection and Processing

For blood collection, animals were restrained gently, the jugular vein area was soaked with povidone–iodine, and approximately 5 mL of blood was drawn using a sterile syringe and needle. The collected blood was divided into two portions; one portion was transferred into a 2 mL EDTA tube for molecular study, and another portion was transferred into a vacutainer tube containing clot activator for serum preparation. Then, samples were quickly brought into the Small Ruminant Research Laboratory, Bangladesh Livestock Research Institute, by maintaining proper cool chain conditions for the required laboratory investigations. Blood samples were centrifuged at 3000 rpm for 10 min to separate serum. Then serum samples were collected in Eppendorf tubes and kept at −20°C and whole blood samples at −80°C before testing in the laboratory (Ayanur et al. 2016; Daif et al. 2022).

### 2.5 Serological Analysis

All sera were tested for the detection of antibodies against the VP7 protein of BTV in goats by competitive enzyme‐linked immunosorbent assay (c‐ELISA), using the ID Screen Bluetongue Competition c‐ELISA kit (ID.vet, France, Ref: BTC‐5P, Lot: O69), following the manufacturer's instructions. The VP7 protein is a highly conserved, group‐specific antigen of BTV, making it an ideal target for serological detection across different BTV serotypes (Belbis et al. 2017; Daif et al. 2022). This c‐ELISA specifically detects antibodies against VP7, allowing reliable identification of BTV exposure in goats. In brief, the optical density (OD) was measured at 450 nm using an ELISA microplate. For each sample, the percentage OD of sample/OD of negative control (S/N percentage) was calculated using the formula provided in the manual: S/N% = (OD_sample_/OD_NC_) × 100. Where OD_sample_ is the OD of the sample, OD_NC_ is the mean value of the OD of the negative control. Samples showing an S/N% less than 40% were observed as positive, whereas those having an S/N% greater than or equal to 40% were observed as negative (Supporting Information File 3).

## 3 Molecular Detection of BTV

### 3.1 Preparation of dsRNA and Amplification of BTV Genome Segment Using Reverse Transcription Polymerase Chain Reaction (RT‐PCR)

Total dsRNA was extracted from ELISA‐positive pooled blood samples (58 pools, each containing 5 of the 290 ELISA‐positive samples), using the PureLink RNA Mini kit (Invitrogen, Thermo Fisher Scientific, USA, Catalog: 12183018A, Lot: 2639227) according to the manufacturer's instructions. After that, extracted RNA was used for PCR or stored at −80°C for further analysis. The following primer sets (Table 1) were used to amplify portions of segments VP7 and NS1 by nested PCR of each segment of target genes. The VP7 and NS1 gene regions were amplified using the one‐step RT‐PCR kit (ABclonal, USA; Catalog No.: RK20404) following the manufacturer's protocol. Configuration for the reaction was as follows: 12.5 µL buffer, 1 µL enzyme mix, 1 µL of each primer (forward and reverse), 4.5 µL nuclease‐free water and 5 µL extracted RNA sample. A PCR tube containing 25 µL of the reaction mixture was used for the process. The nested PCR was done for the second‐round amplification of the 770 and 101 bp PCR products using BTV VP7 gene‐specific primers and BTV NS1 gene‐specific primers, respectively. Five µL of the first‐round amplified product were used as a template for the second round of PCR amplification. Primer details and different conditions of the RT‐PCR method have been described in Table 1. After the PCR reaction, 5 µL of the amplified products were electrophoresed on a 1.5% agarose gel and visualized with ethidium bromide. The generated amplicons were confirmed by their sizes on agarose gel and nested PCR.

**TABLE 1:** Genes, primer sequences and optimal amplification settings for polymerase chain reaction (PCR) assay.

Gene	Primer sequence (5′–3′)	PCR conditions	Amplicon size (bp)	References
BTV‐Seg‐7/VP7	For 1st round F: GTTAAAAATCTATAGAG R: GTAAGTGTAATCTAAGAG	Primary activation at 95°C, 15 min Cycles: 35 Denaturation: 95°C, 1 min Annealing: 39°C, 1 min Extension: 72°C, 2 min Terminal extension: 72°C, 7 min	1156	Tiwari et al. (2000)
	For nested F: ACAACTGATGCTGCGAATGA R: AACCCACACCCGTGCTAAGTGG	All the reaction conditions were the same as for the nested PCR, except the annealing temperature, which was 55°C instead of 39°C	770	
BTV‐Seg‐5/NS1	For 1st round F: GTTCTCTAGTTGGCAACCACC R: AAGCCAGACTGTTTCCCGAT	Primary activation: 95°C, 30 min Cycles: 35 Denaturation at 95°C, 30 s Annealing at 57°C, 30 s Extension at 72°C, 1 min Terminal extension at 72°C, 10 min	274	Ayanur et al. (2016)
	For nested F: GCAGCATTTTGAGAGAGCGA R: CCCGATCATACATTGCTTCCT	Primary activation at 94°C, 5 min Cycles: 40 Denaturation at 95°C, 30 s Annealing at 55°C, 30 s Extension at 72°C, 1 min Terminal extension at 72°C, 10 min	101	

Abbreviation: BTV, bluetongue virus.

### 3.2 Statistical Analysis

The data collected during the survey were entered into Microsoft Excel 2019, checked for quality and coded the data according to the nature of the variables. The data were then converted to a CSV file for analysis of risk factors in STATA 14 (StataCorp, Texas, USA). The numerical variables, including ‘age’ and ‘flock size’, were tested for multicollinearity and showed a non‐linear relationship with each other. Therefore, continuous variables are converted into binary categorical variables by using quantiles, especially medians, to avoid the error of linearity. After that, descriptive statistics were performed to calculate the percentage and 95% CI for each category of individual factor variables. A chi‐square test was applied to determine binary associations of BTV at the animal level. Univariable logistic regression analysis was applied to test the association of individual risk factors with the outcome variables of BTV, and crude odds ratios (CORs), their 95% CI and corresponding *p* values were determined by logistic functions in STATA. For multivariable logistic regression, the backward elimination method was applied to select significant variables. Variables were retained on the basis of statistical significance, biological relevance and absence of multicollinearity. The final model considered only those factors that contributed meaningfully to predicting BTV seropositivity and calculated the adjusted OR (AOR) with CI. The farms were considered a random effect. The *p *< 0.05 was considered significant in the chi‐square test and both univariable and multivariable analyses. The effectiveness of the multivariate logistic regression model was evaluated using the goodness of fit test and the receiver operating characteristic (ROC) curve.

## 4 Results

### 4.1 BTV Seroprevalence in the Study Areas

The BTV‐VP7 antibodies were detected in 290 of 460 sera tested, giving an overall seroprevalence of 63.04% (95% CI: 58.63–67.45). Goats from Jashore district showed the highest seroprevalence, 84.78% (95% CI: 71.13–93.65), whereas Dhaka district had the lowest seroprevalence, 35.42% (95% CI: 22.16–50.54). Univariate logistic regression analysis revealed that BTV seroprevalence varied significantly (*p* < 0.05) among the study locations (*p* < 0.05), and CORs with 95% CI for each district are presented in Table 2 for clarity.

**TABLE 2:** Bluetongue virus (BTV) seroprevalence of goats in the study locations by competitive enzyme‐linked immunosorbent assay (c‐ELISA) by univariate logistic regression analysis.

Locations	Sample tested (positive)	Prevalence (%)	95% CI	COR (95% CI)	*p* value
Thakurgaon	48 (27)	56.25	41.18–70.51	2.34 (1.03–5.33)	0.042
Faridpur	41 (22)	53.66	51.96–85.77	2.11 (0.90–4.95	0.086
Chuadanga	49 (39)	79.59	65.66–89.75	7.11 (2.85–17.71)	<0.001
Bandarban	44 (25)	56.82	41.03–71.65	2.39 (1.03–5.56)	0.041
Kustia	42 (32)	76.19	60.54–87.94	5.83 (2.31–14.70)	<0.001
Gaibandha	42 (20)	47.62	32.04–63.58	1.65 (0.71–3.86)	0.242
Rajshahi	50 (29)	58	43.20–71.81	2.51 (1.11–5.69)	0.026
Jashore	46 (39)	84.78	71.13–93.65	10.15 (3.74–27.5)	<0.001
Dhaka	48 (17)	35.42	22.16–50.54	Ref.	
Sylhet	50 (40)	80	66.28–89.96	7.29 (2.93–18.14)	<0.001
**Total**	**460**	**63.04%**	**58.63**–**67.45**		

Abbreviations: CI, confidence interval; COR, crude odds ratio.

### 4.2 Risk Factors Associated With BTV

#### 4.2.1 Univariable Logistic Regression Model

Table 3 shows the results of an univariable logistic regression study to determine risk variables for BTV infection. However, compared to other goat breeds, cross‐bred goats showed the greatest seroprevalence of BTV infection at 76.16% (95% CI: 69.08–82.32, COR: 3.62), which was statistically significant (Table 3). Adult goats had a significantly greater prevalence of BTV (83.2%, 95% CI: 76.19–89.86, COR: 5.48) than kids (<6 months) and young goats (6–24 months). Seropositivity varied significantly between female goats, poor biosecurity measures, no vector control, no knowledge about BTV and household goat farms, respectively (Table 3). Furthermore, goats with poor BCSs, medium‐sized flocks, free‐ranged reared goats, reared with other species and no PPR vaccination showed higher seropositivity to BTV. But the relationship has not shown any statistical significance.

**TABLE 3:** Univariable logistic regression analysis of risk factors for bluetongue virus (BTV) seropositivity in goats.

Variables	Group	Sample tested (positive)	Prevalence (95% CI)	COR (95% CI)	*p* value
Breed	JP	64 (30)	46.88 (34.28–59.78)	Ref.	
	BBG	224 (129)	57.59 (50.83–64.14)	1.54 (0.88–2.68)	0.130
	Cross	172 (131)	76.16 (69.08–82.32)	3.62 (1.98–6.62)	<0.001
Age (month)	Below 6	59 (28)	47.46 (34.29–60.88)	Ref.	
	6–24	276 (158)	57.25 (50.98–62.94)	1.48 (0.84–2.60)	0.171
	Over 24	125 (104)	83.2 (76.19–89.86)	5.48 (2.74–10.97)	<0.001
Sex	Male	171 (84)	49.12 (41.41–56.86)	Ref.	
	Female	289 (206)	71.28 (65.69–76.42)	2.57 (1.73–3.81)	<0.001
Rearing system	Semi‐intensive	170 (98)	57.65 (49.84–65.17)	Ref.	
	Free range	290 (192)	66.21 (60.44.71.63)	1.43 (0.97–2.12)	0.067
Flock size	Large	169 (100)	59.17 (51.35–66.65)	Ref.	
	Small	121 (75)	61.98 (52.71–70.65)	1.12 (0.69–1.81)	0.629
	Medium	170 (115)	67.65 (60.06–74.61)	1.44 (0.92–2.25)	0.106
BCS	Good	143 (82)	57.34 (48.80–65.56)	Ref.	
	Medium	121 (77)	63.64 (54.40–72.18)	1.30 (0.79–2.13)	0.298
	Poor	196 (131)	66.84 (59.77–73.38)	1.49 (0.96–2.34)	0.075
Housing system	Slat	217 (122)	56.22 (49.34–62.93)	Ref.	
	Floor	243 (168)	69.14 (62.91–74.88)	1.74 (1.19–2.56)	0.004
Biosecurity	Good	208 (108)	51.92 (44.91–58.88)	Ref.	
	Poor	252 (182)	72.22 (66.25–77.65)	2.40 (1.63–3.54)	<0.001
Farm type	Commercial	180 (96)	53.33 (45.76–60.79)	Ref.	
	Household	280 (194)	69.29 (63.51–74.63)	1.97 (1.33–2.90)	0.001
Knowledge about BTV	Yes	116 (57)	49.14 (39.73–58.58)	Ref.	
	No	344 (233)	67.73 (62.50–72.64)	2.17 (1.41–3.33)	<0.001
Vector control	Yes	40 (5)	12.5 (4.18–26.80)	Ref.	
	No	420 (285)	67.86 (63.15–72.30)	14.78 (5.66–38.5)	<0.001
Rearing with other species	No	112 (63)	56.25 (45.56–65.60)	Ref.	
	Yes	348 (227)	65.23 (59.96–70.22)	1.45 (0.95–2.25)	0.088
Vaccination against PPR	Yes	254 (155)	61.02 (54.72–67.05)	Ref.	
	No	206 (135)	65.53 (58.61–72.01)	1.21 (0.83–1.78)	0.319

Abbreviations: BCS, body condition score; CI, confidence interval; COR, crude odds ratio.

#### 4.2.2 Multivariable Logistic Regression Model

**FIGURE 2:**
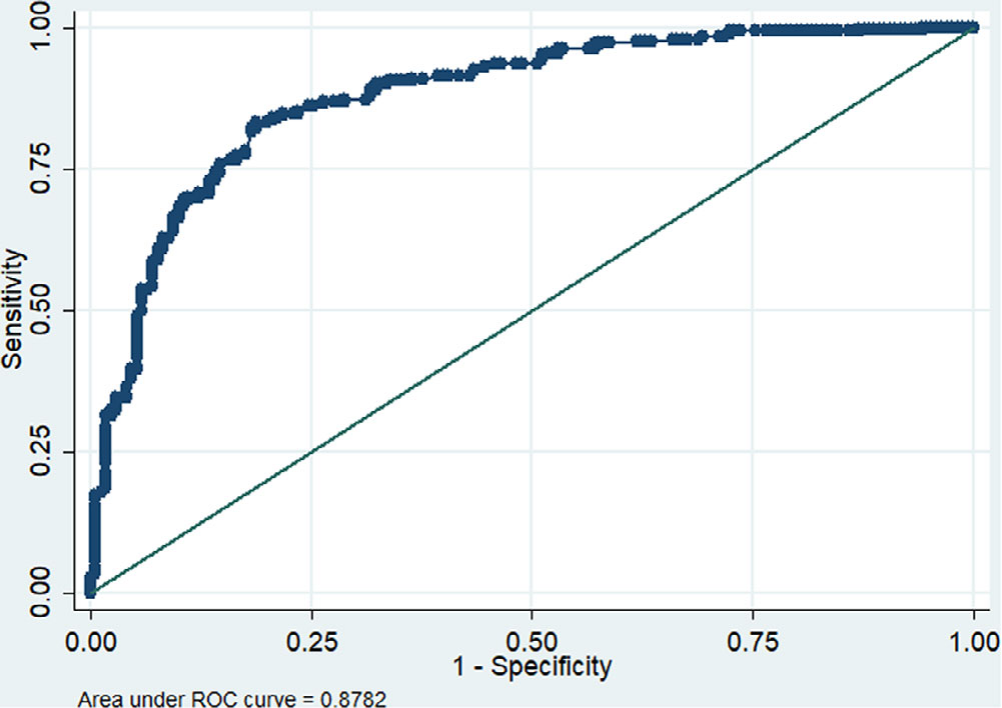
Graph depicting sensitivity against specificity for the ROC curve derived from the final multivariable logistic regression analysis of BTV of goats in Bangladesh. The points of the curve indicate the maximum accuracy of the model.

BTV seroprevalence of goats in Bangladesh was predicted to be influenced by seven variables through multivariate logistic regression analysis: breed, age, sex, flock size, biosecurity, farm types and vector control. The predictive value of these factors has been determined in conjunction with each other. The results of the study showed that the likelihood of testing positive for BTV infection was higher in cross‐breed goats (AOR: 3.4, 95% CI: 1.34–8.63), adult goats over 24 months (AOR: 6.38, 95% CI: 2.42–16.81), female goats (AOR: 1.97, 95% CI: 1.15–3.40), large flock size (AOR: 30.53, 95% CI: 2.91–319.6), household farms (AOR: 33.72, 95% CI: 3.02–375.5) and farms without vector control (AOR: 27.56, 95% CI: 8.60–88.28) (Table 4). Additionally, the goodness of fit test was nonsignificant (*p* > 0.05), indicating that the model adequately fit the data. Moreover, the model demonstrated high predictive accuracy, with an ROC area of 0.8782, confirming reliable differentiation between seropositive and seronegative goats (Figure 2).

**TABLE 4:** Potential risk factors for bluetongue virus (BTV) seropositivity in goat farms of different districts of Bangladesh using multivariable logistic regression analysis.

Variable	Level	Estimates	SEM	Adjusted OR (95% CI)	*p* value
Intercept		−8.28			
Breed	JP			Ref.	
	BBG	1.11	0.44	1.7 (0.71–4.05)	0.226
	Cross	2.11	0.45	3.4 (1.34–8.63)	0.010
Age (month)	Below 6			Ref.	
	6–24	0.38	0.38	1.32 (0.63–2.75)	0.449
	Over 24	1.82	0.48	6.38 (2.42–16.81)	<0.001
Sex	Male			Ref.	
	Female	0.60	0.28	1.97 (1.15–3.40)	0.014
Flock size	Small			Ref.	
	Medium	0.42	0.46	1.69 (0.77–3.67)	0.183
	Large	4.75	1.22	30.53 (2.91–319.6)	0.004
Biosecurity	Good			Ref.	
	Poor	1.60	0.69	26.48 (1.72–405.6)	0.02
Farm type	Commercial			Ref.	
	Household	3.16	1.08	33.72 (3.02–375.5)	<0.001
Vector control	Yes			Ref.	
	No	3.45	0.64	27.56 (8.60–88.28)	<0.001

Abbreviations: BBG, Black Bengal; SEM, standard error of the mean.

### 4.3 Detection of BTV Nucleic Acid by RT‐PCR

**FIGURE 3:**
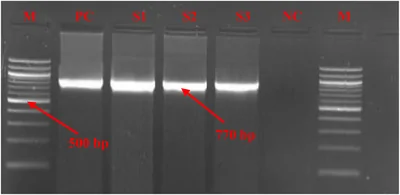
Amplification of the BTV VP7 gene as a product size 770 bp. PC = positive control and NC = negative control; positive samples = S1, S2 and S3 and M = 100 bp DNA ladder.

**FIGURE 4:**
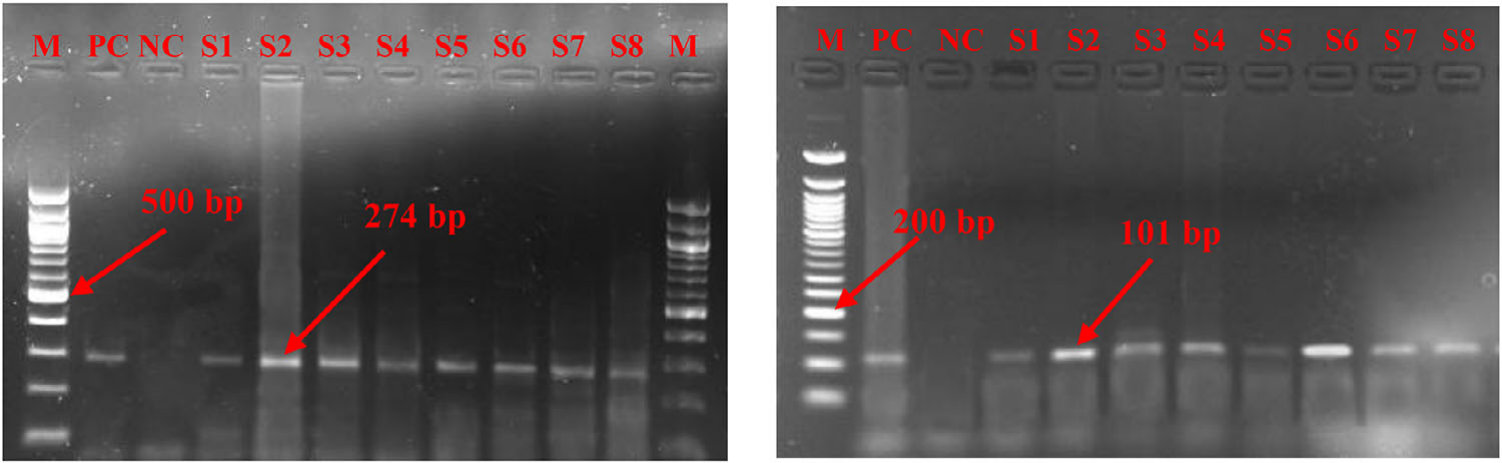
Amplification of the BTV NS1 gene as product size 274 bp (left; M = 100 bp DNA ladder) and 101 bp (right; M = 50 bp DNA ladder). PC = positive control and NC = negative control, positive samples = S1–S8.

In the present study, a nested PCR assay was employed using forward and reverse primers targeting the VP7 and NS1 genes to confirm BTV in ELISA‐positive pooled blood samples from goats. Among the 58 pooled samples (each pool containing 5 of the 290 ELISA‐positive samples), 13 pools (22.4%) tested positive for BTV RNA, confirming the detection of the virus through PCR. By using appropriate primers, PCR products of the predicted sizes were found in the agarose gel electrophoresis at 770 bp for the VP7 gene (Figure 3) and at 273 and 101 bp for the NS1 gene (Figure 4), respectively.

## 5 Discussion

BTV represents a major infectious disease with worldwide impact due to its transmission between international borders via vectors, authorized or unauthorized animal trafficking, or its byproducts (Malik et al. 2018; Bakhshesh et al. 2020). Understanding and monitoring the epidemiological status of BTV infection within a country is crucial for implementing effective measures to control its spread and mitigate the associated socioeconomic impacts (Daif et al. 2022). This study incorporates serological and molecular detection of BTV conducted in goats across different goat‐rich districts of Bangladesh.

The study is the first extensive wide‐scale sero‐epidemiological investigation on BTV in goats from Bangladesh that covers a broad area of previously untouched goat‐prone areas, alongside evaluating its related risk factors. The current investigation employed c‐ELISA for the detection of BTV antibodies, chosen over various serological assays because of a high specificity of 99.96% (Malik et al. 2018). This method has been demonstrated as being reliable and ideal for determining BTV antibodies in sheep, goats and cattle (Malik et al. 2018; Saminathan et al. 2020). The present study reveals an overall seroprevalence of BTV at 63.04%, indicating that over half of the samples tested positive for BTV infection. There are no vaccinations for BT disease given in Bangladesh. The higher level of BTV antibodies signifies that BTV is prevalent in Bangladeshi goats. The result of the present study is nearly similar to Sevik (2023), who reported 63.3% of BTV in goats in Turkey; Bakhshesh et al. (2020) reported a 65.65% prevalence of BTV in goats in Iran; Ishaq et al. (2024) reported 65% in North‐western Pakistan; and Gizaw et al. (2016) reported a 60.33% prevalence of BTV in goats of Ethiopia. However, higher prevalence rates of 85.3%, 77.8%, 78.1% and 70% have been reported by Oryan et al. (2014), El‐Sebelgy et al. (2021), Islam et al. (2023) and Ishaq et al. (2023) in southern Iran, Egypt, Bangladesh and Pakistan, respectively. The contradiction can be attributed to the lower sample size and study area, as well as distinct environmental conditions. High temperatures and vector abundance could be the reasons behind the increasing incidence of BTV, as the virus replicates more effectively above 15°C (Mellor et al. 2000; Malik et al. 2018) and *Culicoides* midges, the primary vectors, are more active in tropical and subtropical regions (Mellor et al. 2000). In our study, the higher prevalence may also be linked to management‐related stressors, including heavy ectoparasite infestation (e.g., ticks), which can weaken host immunity and indirectly enhance susceptibility to viral infections (Karasuyama et al. 2020). On the other hand, our finding is higher than China's 28.1% (Liu et al. 2021), Morocco's 37.5% (Daif et al. 2022), Pakistan's 40.75% (Sohail et al. 2019), Africa's 47% (Medrouh et al. 2024), India's 47.58% (De et al. 2019) and Saudi Arabia's 53.3% (Yousef et al. 2012). Recent evidence indicates that the incidence of BTV and other viral infections may vary among countries, influenced by climate, breed, farming practices and housing conditions (Medrouh et al. 2024; Rahman et al. 2023). This difference may also be due to sheep and cattle, which are the main sources of vectors that can spread disease and make goats that are raised with sheep and cattle more likely to get infected (Schwartz‐cornil et al. 2008; Malik et al. 2018). The situation became worse because of the absence of clinical manifestation of BTV in goats and the lack of routine serological testing, thus failing to detect the ongoing infection (Malik et al. 2018; Saminathan et al. 2020).

The present investigation found a significant association between seroprevalence and breed. Crossbred goats exhibit a significantly higher seroprevalence compared to other breeds (BBG and JP). Small ruminants have been previously reported to have a mild or larger influence on breed susceptibility towards BTV infection (Caporale et al. 2014; Manavian et al. 2017; Sohail et al. 2018). Additionally, some researchers found that Indigenous goats are more resistant to various infectious diseases due to their inherent genetic resistance, and exotic and crossbred goats are more susceptible to many infectious diseases (Bishop and Morris 2007; Kurukulasuriya et al. 2021). Another possible reason for this discrepancy is that the total number of samples analysed from crossbreeds (*n* = 172) was lower than that of natives (*n* = 228), which could have influenced the result.

Age was another significant factor for BTV seropositivity observed in the study. Adult goats had significantly higher seroprevalence compared to other groups of goats. This is most likely due to loss of colostral antibodies, becoming more susceptible with age, the raising of previously unnoticed antibody response, increased vector exposure, or a bigger total surface area of the body in adults compared to young goats (Manavian et al. 2017). Among seropositive goats younger than 6 months, the majority were older than 3 months of age and had already been weaned, indicating that antibody detection in this group predominantly reflects natural exposure rather than passive maternal immunity. The observed seroprevalence primarily reflects BTV exposure in weaned juvenile and adult goats under field conditions.

Another factor significantly associated with seroprevalence was the sex of goats, where females are more often seropositive than males (*p* > 0.05). Similarly, many studies reported that female goats exhibited significantly higher seroprevalence of BTV (Liu et al. 2021; Gaire et al. 2014). This might be because females are reared for longer periods for reproduction, which increases their exposure to *Culicoides* midges. Additionally, lactating females may release higher levels of volatile organic compounds that attract vectors, hence elevating their risk of being bitten by infected *Culicoides* midges (Kelly 2001). In contrast, Sohail et al. (2019) reported higher seroprevalence in male goats compared to females, which might be due to the relatively small number of male goats sampled in their study (*n* = 79) compared to females (*n* = 397).

Flock size was another factor that was significantly associated with seroprevalence. Larger flocks exhibited a higher seroprevalence than small and medium‐sized flocks. This result is consistent with Gaire et al. (2014), who also found more BTV cases in large flocks. It could be due to larger flocks tending to have higher host densities, providing more opportunities for vectors like *Culicoides* midges to feed and transmit the virus (Maclachlan 2011). Additionally, implementing vector control measures (e.g., insecticide application or housing during peak vector activity) is more difficult in large flocks than in small or medium‐sized ones (Saminathan et al. 2020).

The current study revealed significant differences in seroprevalence associated with different farm types and biosecurity measures. There was significantly higher seroprevalence in household farms than in commercial farms, as well as higher seroprevalence observed in farms where poor biosecurity measures were maintained by the farmers. This discrepancy might result from the density of the vector population. Previous studies have documented the geographical variation in Culicoides vector densities with BTV seroprevalence (Gaire et al. 2014; Mellor and Wittmann 2002; Calvete et al. 2006). Additionally, poor adherence to good farming practices and insufficient farmer knowledge of BTV infection in household farms may have influenced this discrepancy. In addition, the vector's sexual and biting activities are facilitated by the poor hygiene of farms, which includes wet soil that is enriched with fresh or decomposed faeces (De et al. 2019).

A significant variation was observed between BTV seroprevalence and the vector control measures implemented by goat farmers. Animals raised without vector control had the highest seroprevalence of BTV. Goat owners lack awareness and knowledge about vector‐borne diseases, specifically BT, which is the main transmission method for BTV (Islam et al. 2023). The complex epidemiological settings of vectors can also influence BTV seroprevalence in the study areas (De et al. 2019; Islam et al. 2023).

PCR‐based methods, including conventional PCR and real‐time quantitative PCR (qPCR), are widely used for detecting BTVs due to their high sensitivity and specificity. These assays target conserved genes like VP7 and NS1, which are critical for reliable diagnosis (Maan et al. 2016; Wilson et al. 2000; Billinis et al. 2001). Here, we first report molecular evidence of BTV in goats in Bangladesh using RT‐PCR techniques. This study used the VP7 gene‐based nested RT‐PCR, a standard gene for BTV detection, to analyse blood samples for the presence of the virus. Conventional PCR targeting the VP7 gene is widely used for detecting BTV due to its conserved nature across all serotypes. The VP7 gene encodes a major core protein essential for group‐specific diagnosis. Studies have demonstrated its reliability and sensitivity in amplifying BTV RNA from various samples, making it suitable for surveillance and outbreak investigations (Rudenko et al. 2019; Anthony et al. 2004). Furthermore, this study applied the NS1 gene‐based nested PCR of BTV using viral RNA of blood samples. This method produced amplified products of 274 and 101 bp, respectively, by using NS1 gene‐specific primers. These results align with Ries et al. (2022), Ayanur et al. (2016), Bhagat et al. (2015), where they used nested PCR using the NS1 gene for BTV identification. The BTV NS1 gene‐based PCR assay serves as a quick, specific and accurate detection tool for analysing clinical field blood samples, facilitating the identification of BT disease and the BTV viral epidemiology (Ayanur et al. 2016).

This study provides valuable baseline information on the seroprevalence, associated risk factors and PCR‐based detection of BTV in goats across different regions of Bangladesh. However, this study also has certain limitations. First, although samples were collected from various farms and districts, flock‐level identifiers were not recorded, and the sampling design was based on individual animals rather than a formal multistage cluster approach. Second, the study focused solely on goats and did not include other susceptible ruminants such as sheep, cattle or buffalo, which may also play a role in BTV transmission. Future studies should incorporate multistage sampling, flock‐level data collection, broader species inclusion and genomic characterization of the circulating BTV serotypes in Bangladesh to support national surveillance and develop effective control programmes.

## 6 Conclusions

In this study, serological testing was used to estimate the nationwide seroprevalence of BT infection and to identify associated risk factors in Bangladesh. Molecular analysis using RT‐PCR further confirmed the active circulation of BTV within this country. The findings indicate that BTV is a serious pathogen with significant economic consequences and is highly prevalent in goats in Bangladesh, despite limited clinical signs being observed. Moreover, goats may pose a potential threat to other ruminant species. To mitigate these risks, farm management practices such as reducing high animal density in herds and avoiding close mixing of susceptible species, along with strict biosecurity measures and the use of insect repellents, are recommended to reduce host–vector contact, decrease infection spread and minimize financial losses in Bangladesh.

## Author Contributions

**Md. Habibur Rahman**: conceptualization, wrote original manuscript, designed the experiment, performed the experiments, data curation, data analysis and interpretation, revised the manuscript. **Md. Nurul Haque**: wrote original manuscript, performed the experiments, data curation. **Md. Zulfekar Ali**: data curation, data analysis and interpretation, critical review and editing. **Lutfun Naher**: performed the experiments, data curation. **Md. Zakir Hassan**: designed the experiment, review and editing. **Sadek Ahmed**: conceptualization, supervision, funding acquisition, project administration.

## Funding

This study was funded by the Bangladesh Livestock Research Institute, Savar, Dhaka, Bangladesh.

## Ethics Statement

The Animal Experimentation Ethics Committee of Bangladesh Livestock Research Institute approved this research project (Reference no.: AEEC/BLRI00102/2023) (Supporting Information File 4). During sample collection, all guidelines for animal care were carefully followed.

## Conflicts of Interest

The authors declare no conflicts of interest.

## Supporting information




**Supporting File 1**: vms370909‐sup‐0001‐SupMat‐File‐1.pdf

**Supporting File 2**: vms370909‐sup‐0002‐SupMat‐File‐2.xlsx

**Supporting File 3**: vms370909‐sup‐0003‐SupMat‐File‐3.xlsx

**Supporting File 4**: vms370909‐sup‐0004‐SupMat‐File‐4.pdf

## Data Availability

The data that support the findings of this study are available in the Supporting Information of this article.
