# Supplementary material for: Sero‐Epidemiology and Molecular Detection of Bluetongue Virus in Goats in Bangladesh
Source: Vet Med Sci. 2026 Mar 31;12(3):e70909. doi: 10.1002/vms3.70909 (PMC13584877; doi:10.1002/vms3.70909)
Supplement: Supplementary file 1 [file VMS3-12-e70909-s002.pdf]

Bangladesh Livestock Research Institute

Savar, Dhaka-1341

Approval No: AEEC/BLRI00102/2023 Date: 25/05/2023

CERTIFICATE OF APPROVAL

This is to certify that the project title "Conservation & improvement of Black Bengal Goat at Bangladesh Livestock Research Institute" is being investigated by Dr. Sadek Ahmed, Principal Scientific Officer & Head, Goat Production Research Division, BLRI et.al has fulfilled the requirements of ethical rules and regulations guided by Animal Experimentation Ethics Committee of Bangladesh Livestock Research Institute to carry-out the project activities. The BLRI AEEC is approving the project after reviewing the protocols and activities under the approval number-AEEC/BLRI00102/2023, Date: 25/05/2023.

Dr. S M Jahangir Hossain
Director General (Current Charge)
&
Convener
Animal Experimentation Ethics Committee of
Bangladesh Livestock Research Institute (BLRI)
Savar, Dhaka
Bangladesh

Phone: +88-02-7791670-2, 7791676, Fax: 7791675, Web: www.blri.gov.bd, E-mail: info@blri.gov.bd, infoblri@gmail.com
