# Supplementary material for: Sero‐Epidemiology and Molecular Detection of Bluetongue Virus in Goats in Bangladesh
Source: Vet Med Sci. 2026 Mar 31;12(3):e70909. doi: 10.1002/vms3.70909 (PMC13584877; doi:10.1002/vms3.70909)
Supplement: Supplementary file 2 [file VMS3-12-e70909-s004.pdf]

বাংলাদেশ প্রাণিসম্পদ গবেষণা ইনস্টিটিউট

Sero prevalence and molecular detection of Bluetongue virus (BTV) in goats of Bangladesh

শীর্ষক প্রকল্পের নমুনা সংগ্রহের ফরম

১. খামারী সম্পর্কিত তথ্য-

(ক) খামারীর নাম: (খ) মোবাইল:

(গ) ঠিকানা: গ্রাম: পোস্ট:

উপজেলা: জেলা:

২. খামারে প্রানী সম্পর্কিত তথ্য-

(ক) খামারের নাম: (খ) খামারের ধরন: পারিবারিক / বানিজ্যিক

(গ) ছাগলের সংখ্যা: (ঘ) জাত: ব্ল্যাক বেঙ্গল / যমুনাপাড়ি / ক্রস

(ঙ) পালন পদ্ধতিঃ মুক্ত ভাবে/ আধা নিবিড় / নিবিড় (চ) ঘরের ধরণ: ফ্লোর / মাচা

(ছ) ছাগল ও ভেড়া একসাথে পালনঃ হ্যা / না (জ) PPR টিকা দেওয়া হয়েছেঃ হ্যা / না

বয়স (<৬ মাস)		বয়স (৬- ২৪ মাস)		বয়স (> ২৪ মাস)	
পু	ম	পু	ম	পু	ম

৩. জীবনিরাপত্তা ব্যবস্থাঃ ভাল / খারাপ (পরিষ্কার-পরিচ্ছন্নতা, অসুস্থ প্রাণী আলাদা রাখা এবং প্রতিরোধমূলক ব্যবস্থার ভিত্তিতে মূল্যায়িত)

৪. রোগ সম্পর্কে ধারণা আছে কি নাঃ হ্যা / না

৫. মশা-মাছি নিয়ন্ত্রণ করা হয় কি নাঃ হ্যা / না

৬. যদি উপসর্গ থাকে তাহলে টিক (√) চিহ্ন দিনঃ

ক) ঠান্ডা খ) শ্বাস কষ্ট গ) নাকে পানি ঘ) মুখে ঘা/আলসার ঙ) গর্ভপাত

চ) লালা পড়া ছ) পা খোঁড়ানো/ করোনারি ব্যান্ডে ঘা জ) জিহবা নীল রং

৭. নমুনা সম্পর্কিত তথ্য-

(ক) নমুনার ধরন: রক্ত / সিরাম / প্লাজমা (খ) প্রানীর ট্যাগ নং: (গ) বয়স:

(ঘ) লিঙ্গা: পুরুষ / মহিলা (ঙ) BCS: ভাল / মধ্যম / খারাপ

তথ্য দানকারীর নাম ও স্বাক্ষর, **তথ্য গ্রহণকারীর নাম, স্বাক্ষর, তারিখ:**

Bangladesh Livestock Research Institute

Sero prevalence and molecular detection of Bluetongue virus (BTV) in goats of Bangladesh

Sample Collection Form

1. **Farmer related information**

(a) Farmer's Name: (b) Mobile:

(c) Address: Village: Post office:

Upazila: District:

2. **Information about farm animals** (where needed, please use put √ mark)

(a) Name of the Farm: (b) Type of farm: Household /Commercial

(b) Number of Goats: (c) Breed: BBG / Jamunapari / Cross

(d) Rearing System: Free / Semi-intensive (e) Housing System: Floor / Slat

(f) Rearing with other species: Yes / No (g) PPR vaccination: Yes / No

**Age (<6 months)**		**Age (6-24 months)**		**Age (>24 months)**	
Male	Female	Male	Female	Male	Female

3. **Biosecurity status:** Good / Poor (based on cleaning, isolation, and preventive practices)
4. **Knowledge about BTV:** Yes / No
5. **Vector control measures:** Yes / No
6. **Signs and Symptoms:** (where needed, please use put √ mark)

i) Cold and Fever ii) Dyspnea iii) Nasal discharge iv) Oral erosions

v) Abortion vi) Salivation vii) Lameness/coronitis viii) Tongue cyanosis

7. **Sample Related Information** (where needed, please use put √ mark)

(a) Type of sample: Blood / Serum (b) Tag No.: (c) Age:

(d) Sex: Male / Female (e) BCS: Good / Average / Poor

Signature of the Respondent

Name & Signature of the Interviewer
